# Constructing synthetic populations in the age of big data

**DOI:** 10.1186/s12963-023-00319-5

**Published:** 2023-10-31

**Authors:** Mioara A. Nicolaie, Koen Füssenich, Caroline Ameling, Hendriek C. Boshuizen

**Affiliations:** 1 Centre for Nutrition, Prevention and Health Services, RIVM (National Institute for Public Health and the Environment), P.O. Box 1, Mailbox 86, 3720 BA Bilthoven, The Netherlands; 2 Capaciteit Orgaan (Advisory Committee on Medical Manpower Planning), Mercatorlaan 1200, 3525 BL Utrecht, The Netherlands

**Keywords:** Synthetic population, Disclosure risk

## Abstract

**Background:**

To develop public health intervention models using micro-simulations, extensive personal information about inhabitants is needed, such as socio-demographic, economic and health figures. Confidentiality is an essential characteristic of such data, while the data should reflect realistic scenarios. Collection of such data is possible only in secured environments and not directly available for open-source micro-simulation models. The aim of this paper is to illustrate a method of construction of synthetic data by predicting individual features through models based on confidential data on health and socio-economic determinants of the entire Dutch population.

**Methods:**

Administrative records and health registry data were linked to socio-economic characteristics and self-reported lifestyle factors. For the entire Dutch population (*n* = 16,778,708), all socio-demographic information except lifestyle factors was available. Lifestyle factors were available from the 2012 Dutch Health Monitor (*n* = 370,835). Regression model was used to sequentially predict individual features.

**Results:**

The synthetic population resembles the original confidential population. Features predicted in the first stages of the sequential procedure are virtually similar to those in the original population, while those predicted in later stages of the sequential procedure carry the accumulation of limitations furthered by data quality and previously modelled features.

**Conclusions:**

By combining socio-demographic, economic, health and lifestyle related data at individual level on a large scale, our method provides us with a powerful tool to construct a synthetic population of good quality and with no confidentiality issues.

## Introduction

Given the rise in computing power, micro-simulation models of populations are increasingly used to support decision-making in policy and fields such as epidemiology, demography, urban and environmental modelling (e.g. [6, 8, 9]).

An important part of individual based modelling is the construction of a starting population for the simulation [3]. Next to being a starting point, this plays a role in calibrating individual transition probabilities against population marginals [5]. In this starting population the marginal and joint distributions of relevant variables, such as baseline demographic variables, should be descriptive of the real-world population as good as possible. Due to the increased digitalisation of society, more and more information is collected in digital form on all population members, and statistical offices can increasingly exchange this information and link it to other data collection forms such as surveys. This information can be used to construct more accurate starting populations for modelling that do not depend on conditional independence assumptions that are implicit in some of the methods used before (e.g. [10]).

An important limitation of the availability of large amounts of information on individuals in a population is that such data constitute sensitive information, making privacy a primary issue. For open use, the disclosure risk of such data needs to be low, while the statistical structure should be as realistic as possible. Although in many countries data are available for selected researchers, this is usually under strict conditions in order to minimize the risk of disclosure of information on each single individual. Running a model on real population data therefore generally will only be allowed within the secure environment (computer system) of the trusted party that manages these data. Running simulation models within these systems is generally not very practical, for instance due to lack of portability of software or lack of sufficient computer power. Furthermore, in the spirit of open science, it is preferable to deliver micro-simulation models, including their data, as a public resource widely available.

Construction of a synthetic population to be used outside a secured environment can overcome these difficulties. Such a population will reflect the structure of the available population data, but it will not represent real persons, though persons are constructed from the available population. In the remaining of this paper, such population will be referred to as synthetic population. Most papers on constructing synthetic populations focus on taking multiple random draws of individuals owning detailed personal information from a smaller size survey sample in order to obtain a larger synthetic population [1, 2, 11], where the population constructed should be consistent with known demographics. Other approaches encompass synthetic reconstruction [19], combinatorial optimization [18] and model-based generation. The task in the first approach (upsampling smaller surveys) is to find inclusion probabilities for the surveyed individuals that deliver a weighted sample with equal marginal frequencies to those available from demographic sources. These methods largely overlap with methods for the construction of survey weights that are used to make survey statistics representative for those of a target population. Others use simulation of past historical data, where the micro-simulation is run from the birth of each individual (a moment in the past) to deliver current prevalence of diseases and risk factor histories [10].

This paper presents another methodology, in which regression models fitted on the original linked data are used to generate a synthetic population. We illustrate this for the case of a Dutch population targeted at chronic disease modelling. In chronic disease modelling, one needs a starting population with known socio-demographic characteristics, exposures to risk factors of diseases and presence of chronic diseases.

The remainder of this paper is organized as follows: “Material and methods” section provides the description of our method. “Results” section provides the evaluation of our method and “Discussion” section concludes the paper. “Appendix 1” introduces the variables used and “Appendix 2” illustrates the regression models used.

## Material and methods

The goal of the method is twofold: one, the protection against disclosure risk for individuals that make up the original dataset and second, to extract the available information from the original data for modelling purposes. Therefore, the synthetic population in constructed in a two-step procedure. The first step is carried out on microdata within the computer system of Statistics Netherlands (SN), under strict conditions regarding privacy and it boils down to constructing predictive equations that no longer contain information which can be traced back to a particular individual. In the second step these equations are used to drawn a synthetic population outside the microdata environment.

The predictive equations in the first step were constructed as follows: starting from the original data set, a designated set of variables was selected with no identity disclosure risks: age, gender, region of residence (so-called COROP code) and level of urbanization, further referred to as the "seed variables". Their role is, among others, to create structure in the population, such as by means of stratification. Following the Statistics Nederland guidelines on disclosure risk, a requirement was to use a stratum sample size of minimally ten persons, that is, ten persons sharing the same combination of seed variables. Although there were a couple of strata with a lower number of individuals, the requirement was waved due to the insensitivity of the disclosed information (age, gender and residence). The number of individuals in each stratum was recorded and exported from the microdata environment for later stages of our approach.

Within the microdata environment, the seed variables were used as starting point to build regression models in a variable-by-variable approach, for a set of designated, confidential variables, such as socio-demographics, lifestyle and presence of diseases. The estimated model parameters as well as their covariance matrices were also recorded and exported outside the microdata environment.

In the second step, the frequencies of the seed variables strata and the estimated predictive equations were used, both outside the microdata environment, for the generation of a synthetic population.

In the following paragraphs, each step is presented in more detail. “Appendix 1”, Table 5 illustrates the order in which the variables were included in the sequential modelling process, as well as the sources of these variables.

### Data sources

The targeted population is the population of the Netherlands at the 31st of December 2012, comprising 16 778 708 individuals (population size of the Netherlands on December 31st 2012). Our data sources were individual level, non-public, linkable microdata sets of Statistics Netherlands, made available under strict conditions regarding privacy issues. Virtually complete data were available on date of birth, gender, marital status, region of residence, level of urbanization, ethnicity, percentile group of household capital, source of income, percentile group of household income and household composition. Incomplete data on the highest achieved level of education were available for the non- institutionalized Dutch population aged 15 or older (see “Appendix 1”, Table 5).

Self-assessment of smoking, BMI and physical activity level were available for a sample of non-institutionalized individuals older than 18 years from survey data collected within the Dutch Public Health Monitor (DPHM) 2012 [4, 16]. The sample comprises 387 195 participants (3.0% of the Dutch population, proportionally sampled). For this edition of the monitor, nation-wide harmonized health surveys were conducted by the 28 Municipal Dutch Health Services and Statistics Netherlands on 415 municipalities comprising questions on self-reported health, health perception and health-related behaviours of persons aged 19 years and older. The average participation rate was 47%. A secured identification number was given to each participant. It was therefore possible to link the DPHM with registry data at individual level within the secured environment of Statistics Netherlands. Several Municipal Health Services oversampled the elderly or those living in deprived areas, so that the sample as such is not representative for the Netherlands. However, the fitted models condition on age and socio-economic factors, this is partially mitigated.

The issue of missing data in the targeted variables of the Dutch Health Monitor 2012 due to the person-level non-response was addressed by means of multiple imputation, using the multivariate chained equation approach implemented in the mice R package [15]. Five replications of complete risk factors data were implemented leading to the creation of five sets of regression coefficients. The latter were pooled using standard multiple imputation rules [13].

Individual probabilities of having coronary heart disease (CHD), stroke, diabetes or chronic obstructive pulmonary disease (COPD) in 2012 were calculated from prediction models using demographic data and data on drug reimbursement. These prediction models were developed using as outcomes individual data on hospital admission and primary care use. In short, the construction of the prediction model involved using LASSO regression model for variable selection followed by a regular regression, as described in Füssenich et al. [7].

Incidence of lung and pancreatic cancer were available from the Netherlands Comprehensive Cancer Organization cancer register (IKNL), which records all individual cancer diagnoses in the Netherlands.

### Statistical prediction models

Taking a sequential approach, a series of prediction models are built as follows: for each newly targeted variable, a prediction model is fitted using as predictors only the seed variables and the variables included in preceding prediction models. The approach is initiated with a regression model for the first outcome variable which uses as predictors only the seed variables. So forth, for the *k*-th outcome variable, the predictors were the seed variables and the outcome variables ranging from 1 to k − 1; until the list of outcomes is exhausted.

This approach has the potential to ensure accurate statistical properties (e.g. to preserve the moments of distribution and the associations between variables) for the selected confidential variables, if the fitted models capture the distributions and associations correctly. However, models are always limited and bias might be introduced when the models do not capture all relevant relations or distributions. The sequential nature of the procedure implies that the inaccuracies in the prediction resulting from the first model will cause inaccuracies in all subsequent predictions. In order to optimize the predictions, the method uses with priority the variables available for the entire Dutch population, and within this set a ranking top-down is made following the decrease in magnitude of correlation among variables, ranking which dictates the order in the modelling sequence.

As such, the first modelled variable is the main income source of the household, being the main driver of the social heterogeneity in the population, using the population defined in the configuration of the seed variables. Next two outcomes modelled were spendable household income and household capital, respectively, knowing that these can vary dramatically with the source of income. The predictive equations are subsequently extended by introducing successively type of household, household size and ethnic group. The reason for the introduction of the education variable rather late in the sequential modelling approach is the fact that it was the only variable of the nation-wide registry data with a large amount of missingness (more than 40%, with missingness strongly dependent on age).

After the nation-wide variables—with the exception of the presence of cancer—health and lifestyle information from the Dutch Health Monitor sample was added, such as BMI, smoking status and physical activity. Taking into account that lifestyle factors recorded in the Dutch Health Monitor sample were highly correlated with the two types of cancer, separated models for pancreas and lung cancer presence were subsequently fitted (in this order) using data on the entire Dutch population. In modelling these cancer types, lifestyle factors were accommodated by means of the missing indicator method [14], seen that only 2% of the whole Dutch population participated in the Dutch Health Monitor study.

Given that diseases such as CHD, stroke, diabetes and CODP could be consequences of exposure to risk factors, they are modelled in this order in the remaining of the sequential procedure. These variables were expressed not as self-reported diagnosis registered in the Dutch Health Monitor study, but as predicted probabilities from an earlier model (see [7]), where the predictions were largely driven by the use of particular pharmaceutical drugs (a nation-wide available variable). Though these variables were available for the whole Dutch population, they were used in models fitted on the Dutch Health Monitor sample only.

In “Appendix 1”, the details of all these fitted models are reported. Following the observation that many variables varied by age in a non-linear fashion, spline functions of age were employed to represent these relationships. As several variables were associated with gender, all models were stratified by gender and, when necessary, by other variables. As a general feature, the main effects only approach is taken in model building.

### Construction of initial population

From the population in the configuration of seed variables exported from the confidential environment the synthetic population of size 16,768,952 (after listwise removal of participants with missing data) was constructed by randomly drawing from the predictive equations as follows. The first predictive equation was applied on the seed population and generated the first synthetic variable. Notably, these generated values do not contain real data for the targeted variable configuration. Next, a sequential simulation from the subsequent predictive equations was performed. The construction was limited to the ages 0–105 years, as this age range is generally accepted to be included in simulation models.

### Methods of evaluating the results

As a method to investigate the utility of our approach, the statistical attributes of the synthetic population were compared to those of the confidential original population using univariate and multivariate statistics. For the univariate results, there were used the generated frequencies for categorical variables and the first four moments of risk factors distribution for continuous variables. For the multivariate results, there were used joint distributions stratified by age class, gender, smoking status and educational level. For this stratification, age was recoded as an eight-level categorical variable (from 20 to 80 years in 10-years age classes, with two extra categories for younger than 20 years and older than 80 years).

## Results

Univariate results based on the original confidential population and the synthetic population are reported in Table 1, as frequencies of all categorical variables and in Table 2, as the first four moments of continuous variables.

**Table 1:** Frequencies (in per cent) of categorical variables in the original confidential population and in the synthetic population

Variables	Original confidential population	Synthetic population
	Frequencies	Frequencies
*Lung cancer*		
No	99.86	99.88
Yes	0.14	0.12
*Pancreas cancer*		
No	99.99	99.99
Yes	0.01	0.01
*Main source of income*		
Employee	47	47
Civil servant	7.4	7.4
Salary as company director	2.4	2.4
Other income from labour	0.3	0.3
Income as company owner	14.7	14.7
Income from property	0.4	0.4
Unemployment benefits	1	1
Disability pension	2.9	2.9
Retirement pension	17.8	17.8
Social assistance benefits	3.2	3.2
Other social security	1	1
Study grant	0.8	0.8
Other	0.1	0.1
No income	1	1
*Household size (number of persons)*		
1	17.5	17.2
2	29.7	30.2
3	16.4	16.7
4	23.1	22.4
5	9.4	9.4
6 and more	3.9	4.1
*Migration background*		
Dutch	78.9	78.7
Moroccan	2.2	2.2
Turkish	2.4	2.4
Surinam	2.1	2.1
Netherlands Antilles and Aruba	0.9	0.9
Other non-Western	4.2	4.2
Other Western	9.4	9.5
*Type of household*		
Institutional	1.4	1.6
Non-institutional	98.6	98.4

* Hospital admission data from the National Medical Registry (LRM), primary care data from the Netherlands Institute for Health Services Research (NIVEL), drug reimbursement data from the Dutch registry on medication use (Medicijntab), all provided through Statistics Netherlands

**Table 2:** Summary statistics of continuous variables in the original confidential population and in the synthetic population

Variables	Original confidential population				Synthetic population			
	Mean	Standard deviation	Skewness	Kurtosis	Mean	Standard deviation	Skewness	Kurtosis
Property	50.49	30.18	− 0.11	− 1.25	49.76	29.67	− 0.04	− 1.24
Income	59.43	27.39	− 0.44	− 0.85	58.41	27.85	− 0.43	− 0.93
Age^a^	40.29	22.96	0.075	− 0.93	40.29	22.96	0.075	− 0.93

^a^The original population contained 9756 subjects not included in the synthetic population due to missing data

In general, the frequencies of the synthetic data are close to those of the original data, although for the number of persons in the household and type of household the numbers were slightly different between synthetic and original. For lung cancer the prevalence was slightly lower in the synthetic population.

To get an impression on the behaviour of the lifestyle variables for people older than 18 years, which were only available for a sample of the population, in Tables 3 and 4 these were compared between the synthetic data and the Dutch Health Monitor sample. However, this comparison should be made with caution as the latter is not a representative sample, as illustrated by the differences in age reported in Table 4. This also drives much of the differences seen in disease probabilities in Table 3. The proportion of smokers in the synthetic population (22.4%) of age 19 and older is lower than the 22.8% reported from the Dutch Health Monitor sample for the same period [12].

**Table 3:** Summary statistics of categorical variables in the original confidential Dutch Health Monitor sample and in the synthetic population for individuals aged 18 +

Variables	DPHM (age > 18)	Synthetic population (age > 18)
	Frequencies	Frequencies
*Smoking*		
Never smoker	41.6	26.9
Past smoker	41.4	50.7
Light smoker	13.3	16.3
Heavy smoker	3.7	6.1
*Physical activity (complies with norms)*		
No	34.1	37.1
Yes	65.9	62.9
*Education (completed) [SOI level]*		
Primary or less [1,2]	12.8	8.4
Lower secondary [3–6]	18.8	13.5
Higher secondary [7–10]	41.8	53.3
Lower Tertiary [11–13]	18.2	16.3
Higher tertiary [14+]	8.4	8.3
Diabetes present	11.5	5.8
COPD present	6.6	7.9
CHD present	10.5	2.7
Stroke present	5.4	4.4

**Table 4:** Summary statistics of continuous variables in the (confidential) Public Health Monitor sample and in the synthetic population for individuals older than 18 years

Variables	DPHM sample (age > 18)				Synthetic population (age > 18)			
	Mean	Standard deviation	Skewness	Kurtosis	Mean	Standard deviation	Skewness	Kurtosis
Age	57.06	17.88	− 0.34	− 0.79	48.98	17.91	0.22	− 0.76
BMI	25.73	4.09	0.87	1.43	25.71	4.06	− 0.04	0.13
Property	60.20	28.39	− 0.56	− 0.79	51.25	29.67	− 0.10	− 1.23
Income	58.10	25.89	− 0.26	− 0.94	56.57	28.00	− 0.35	− 1.00

Note that the composition of the survey sample differs from that of the whole Dutch population

Comparing the moments, we see that the standard deviation of BMI is similar in the synthetic population and in the original data. However, skewness and kurtosis differ. As the model used assumes normally distributed residuals, skewness and kurtosis of the generated data are close to zero, while they are larger for the original data.

**Fig. 1:**
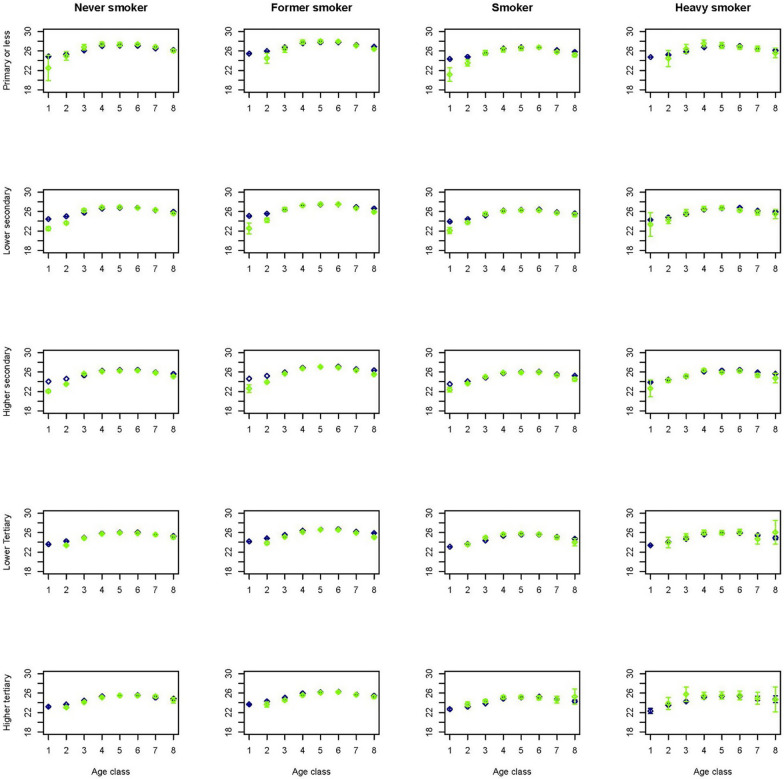
Estimated BMI mean and corresponding 95% CI for men in each 10-year age class (1 =  < 20, 2 = 20–29, 3 = 30–39, 4 = 40–49, 5 = 50–59, 6 = 60–69, 7 = 70–79, 8 = 80 +) by smoking status and educational level in the synthetic population (blue) and in the confidential original data from the DPHM survey (green)

**Fig. 2:**
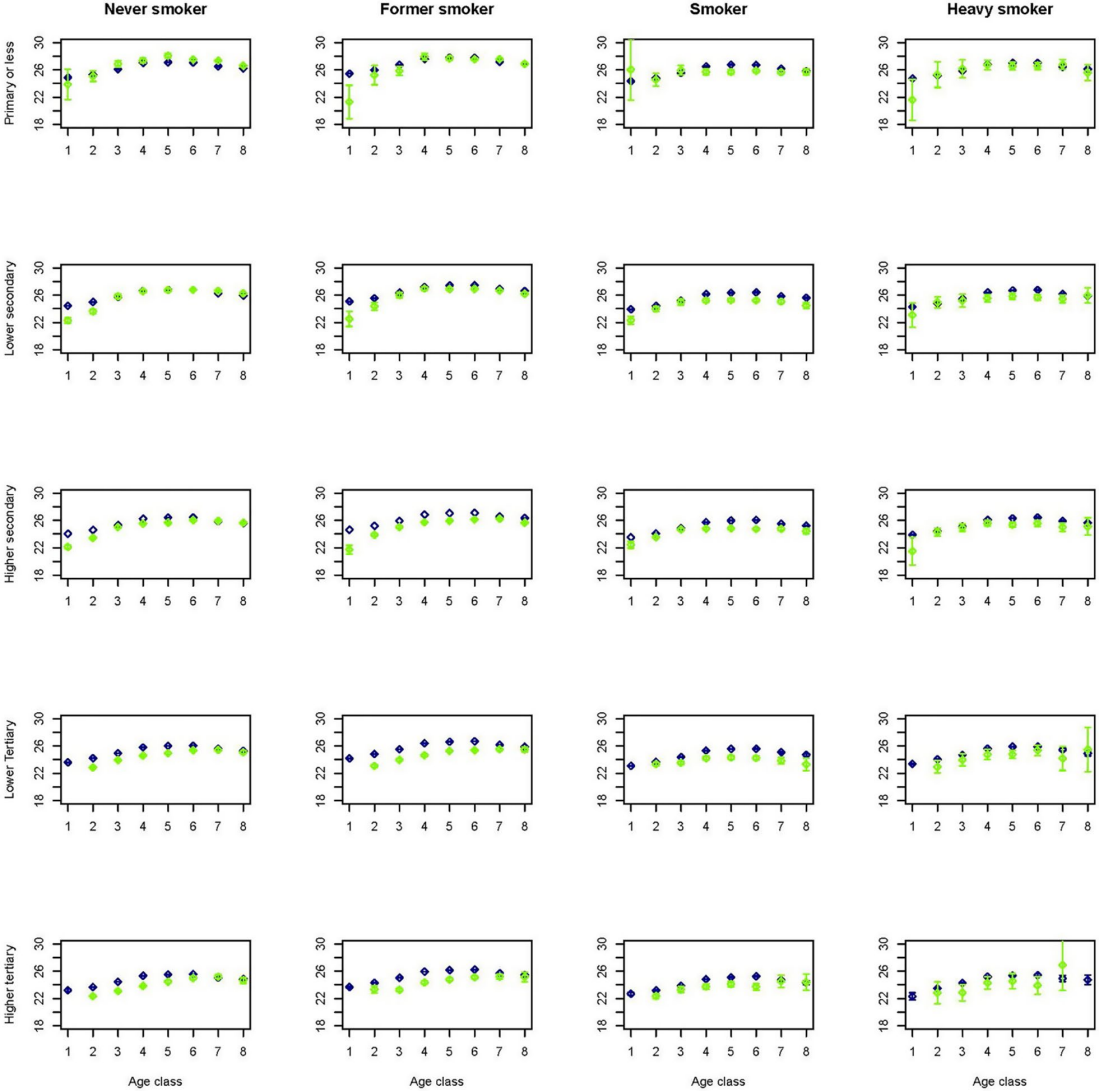
Estimated BMI mean and corresponding 95% CI for women in each 10-year age class (1 =  < 20, 2 = 20–29, 3 = 30–39, 4 = 40–49, 5 = 50–59, 6 = 60–69, 7 = 70–79, 8 = 80 +) by smoking status and educational level in the synthetic population (blue) and in the confidential original data from the DPHM survey (green)

**Fig. 3:**
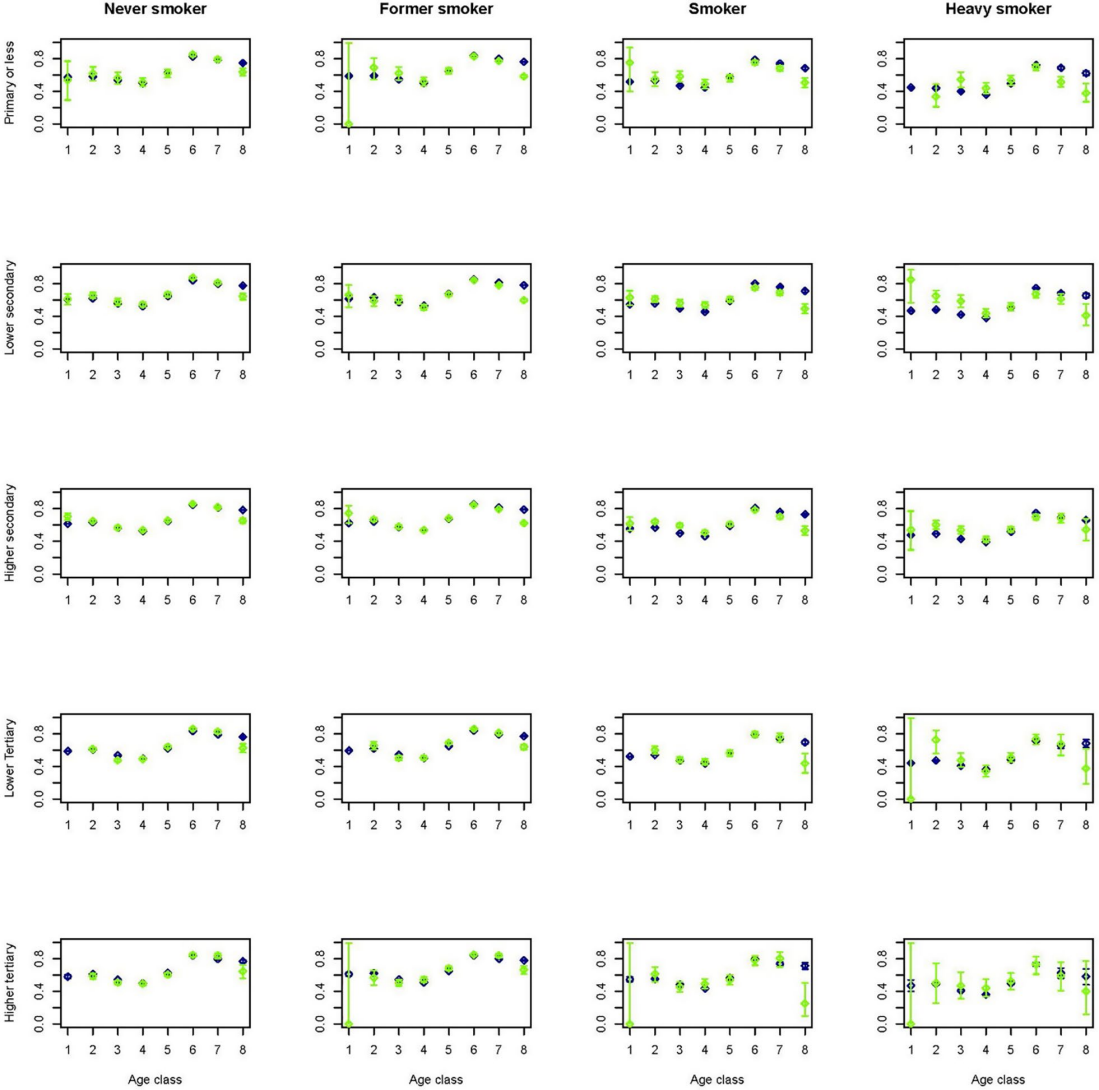
Prevalence of being sufficiently physical active and corresponding 95% CI for men in each 10-year age class (1 =  < 20,2 = 20–29, 3 = 30–39, 4 = 40–49, 5 = 50–59, 6 = 60–69, 7 = 70–79, 8 = 80 +) by smoking status and educational level in the synthetic population (blue) and in the confidential original data from the DPHM (green)

**Fig. 4:**
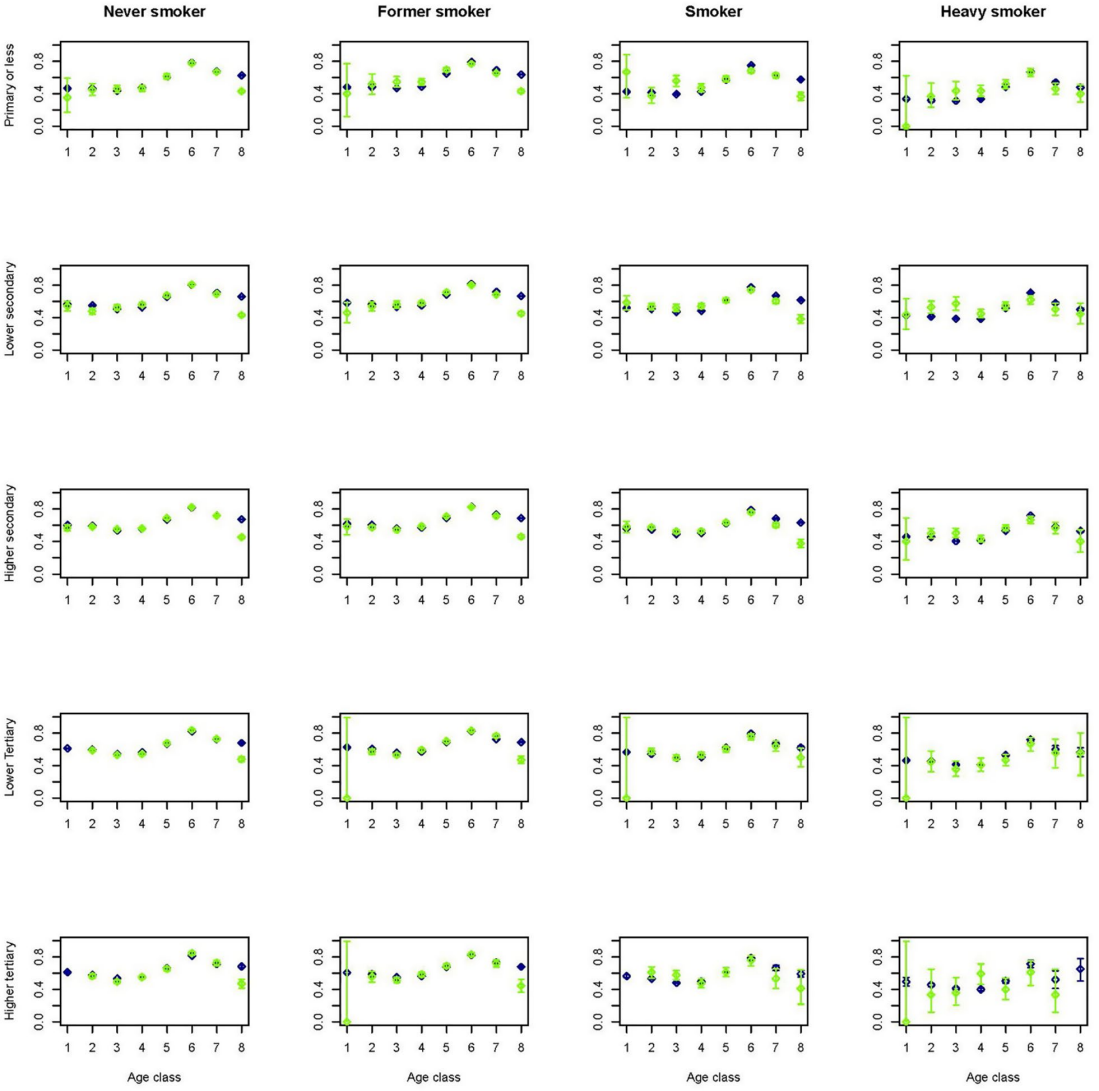
Prevalence of being sufficiently physical active and corresponding 95% CI for women in each 10-year age class (1 =  < 20,2 = 20–29, 3 = 30–39, 4 = 40–49, 5 = 50–59, 6 = 60–69, 7 = 70–79, 8 = 80 +) by smoking status and educational level in the synthetic population (blue) and in the confidential original data from the DPHM (green)

To get an idea on how the predictions performed in a multivariate setting, figures for a selection of outcome variables are created stratified by age, gender, smoking and educational group. As smoking status is available for the Dutch Health Monitor sample only, again these figures contrasted the prevalence in the synthetic population with that in the Dutch Health Monitor sample. This multivariate approach comes with the advantage of displaying more accurate relationships due to the fact that by stratification variation due to the set of variables in strata is controlled for. Figures 1 and 2 indicate that in the synthetic population the mean BMI seems to be overestimated in lower age groups and in women with lower or higher tertiary education. Figures 3 and 4 indicate that mean physical activity is generally correctly estimated in the synthetic population compared to the Dutch Health Monitor sample with the exception of the 80+ sample, where it was estimated to be higher.

**Fig. 5:**
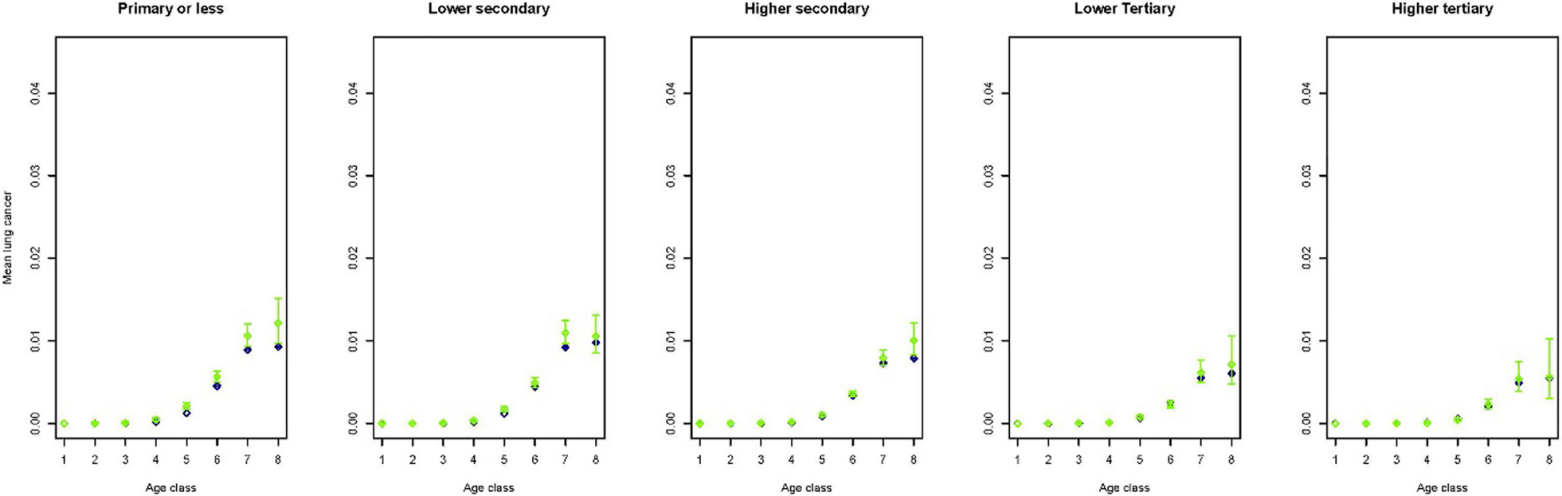
Estimated mean of lung cancer presence and corresponding 95% CI for men stratified by education level and across 8 age classes (1 =  < 20, 2 = 20–29, 3 = 30–39, 4 = 40–49, 5 = 50–59, 6 = 60–69, 7 = 70–79, 8 = 80 +) in the synthetic population (blue) and in the confidential original data of the DPHM survey linked to the cancer registry (green)

**Fig. 6:**
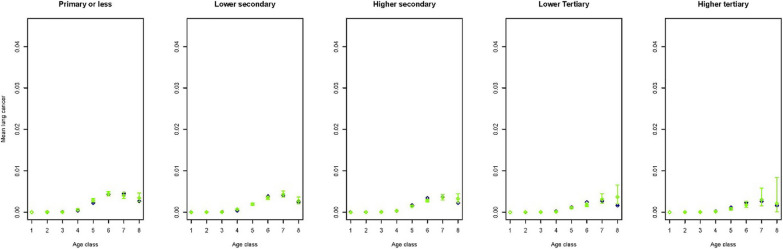
Estimated mean of lung cancer presence and corresponding 95% CI for women stratified by education level and across 8 age classes (1 =  < 20, 2 = 20–29, 3 = 30–39, 4 = 40–49, 5 = 50–59, 6 = 60–69, 7 = 70–79, 8 = 80 +) in the synthetic population (blue) and in the confidential original data of the DPHM survey linked to the cancer registry (green)

Figures 5 and 6 show that lung cancer prevalence is slightly lower in the lower educated in the synthetic population than in the DPHM sample.

**Fig. 7:**
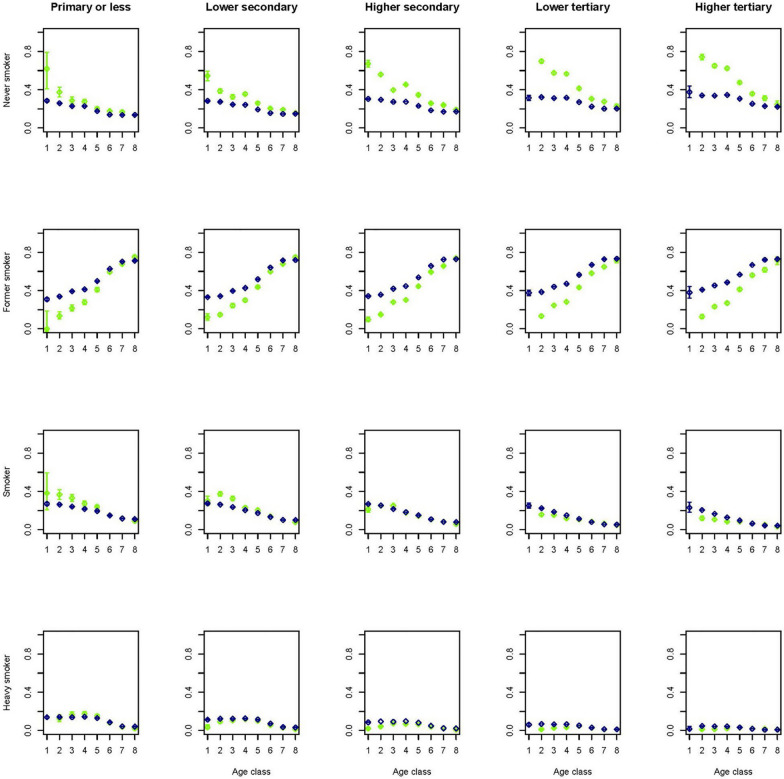
Percentage of smoker categories across 8 age classes (1 =  < 20, 2 = 20–29, 3 = 30–39, 4 = 40–49, 5 = 50–59, 6 = 60–69, 7 = 70–79, 8 = 80 +) and stratified by education level for men in the synthetic population (blue) and in the original confidential data of the DPHM survey (green)

**Fig. 8:**
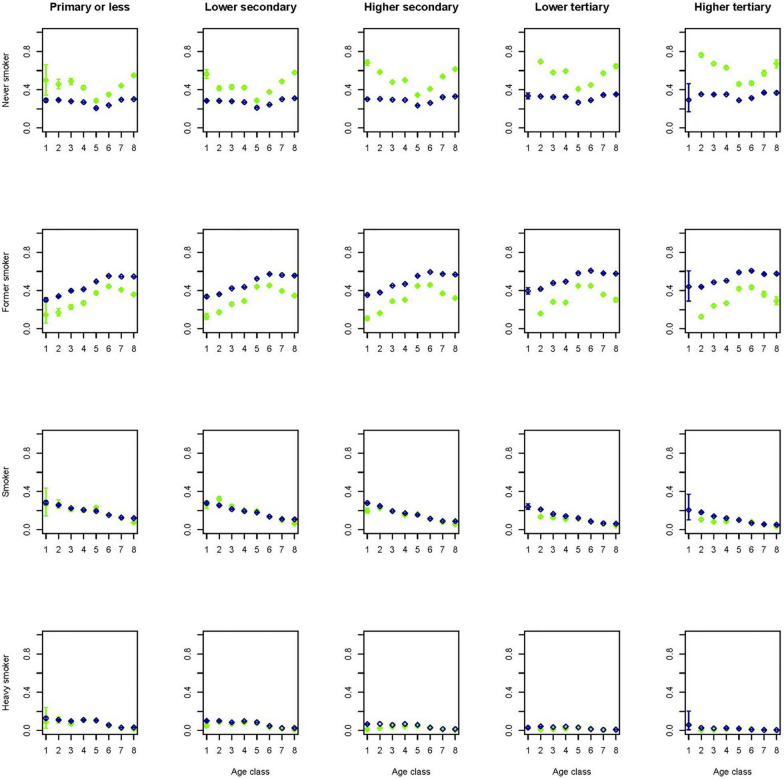
Percentage of smoker categories across 8 age classes (1 =  < 20, 2 = 20–29, 3 = 30–39, 4 = 40–49, 5 = 50–59, 6 = 60–69, 7 = 70–79, 8 = 80 +) and stratified by education level for women in the synthetic population (blue) and in the original confidential data of the DPHM survey (green)

Figures 7 and 8 show that smoking prevalence seems to be reconstructed reasonably well, but the separation of non-smokers into former smokers and never smokers in the younger age groups differs considerably from the data in the Dutch Health Monitor. In the smoking model, demographic variables such as ethnicity and region of residence have relatively large coefficients and might be differently distributed in the general population compared to the Dutch Health Monitor sample. For instance, those with a non-western background comprised 4.5% of the Dutch Health Monitor sample, while as much as 11.8% of the whole Dutch population.

## Discussion

The objective of this paper is to develop a methodology for the generation of a synthetic population for application in chronic disease modelling, based on linked data at the level of individuals, household’s demographics and lifestyle characteristics in the presence of disclosure risk. A synthetic population amounting to the size of the total Dutch population of 2012 was created with realistic characteristics. Our method relies on the construction, in a sequential manner, of regression models for the distributions of individual attributes conditional on a set of determinants. It then uses these regression models to simulate a population by drawing from them. Each synthetic individual has a probabilistically assigned value for all but the initial determinants age, gender, region of residence and level of urbanization, the latter being used as in the original data.

An important part of our data were virtually complete at the individual level (household size and income information based on Tax Authority data, demographic and property information). This yields, on the one hand, the benefit of dealing with minimum uncertainties in the modelling of these variables. On the other hand, model selection was time consuming, as the choice of potential models is large and running times on 16 million records are substantial, specifically when using complex models. As our approach needs parametric models, more automated prediction methods like random forest or other ensemble machine learning approaches could not be used. We settled for relatively simple models, assuming for instance normal residuals and only including a minimum of interaction terms by means of stratification.

This means that the generated data reflected these assumptions, and the generate BMI data are close to being normally distributed, while in the original data they were not. Whether this is a problem will depend on how the synthetic population is further used. For uses as an initial population in micro-simulation of disease we do not deem this problematic. If the skewness of the original data needs to be reflected, the method can be easily adapted by using a model that better reflects such a feature of the data.

An analysis of the goodness-of-fit of the synthetic to the true population using summary statistics and qq-plots showed that it was possible to achieve a high degree of accuracy for the set of first 7 variables (from age to ethnic group), given the availability of the true, complete population. For the next variables, based on the Dutch Public Health Monitor sample, validation of the results was difficult, as this sample displays a different composition than the whole Dutch population. For instance, in the synthetic population the number of former smokers was considerably higher and the number of never smokers lower than in the Dutch Public Health Monitor sample, despite the latter being younger. This means that the Dutch Public Health Monitor sample has a composition that deviates importantly from the general population. Therefore, the stratified comparisons we conducted are more informative. These comparisons depict several similarities, but some disparities were noted, which are probably due to insufficient use of interaction terms in the modelling. Further validation could be done using information from other sources [17], or by using survey weights on the original data. This is especially valuable where comparison with the confidential original data is not very meaningful, as is the case for the data contained in the Dutch Public Health Monitor.

For prevalence of lung and pancreas cancer, we had population wide data, but models were partly dependent on variables (like smoking) that came from the Dutch Public Health Monitor sample, covering only 2% of the whole population. Lung cancer prevalence was slightly lower than it should be. This might be due to the way the model was constructed, which is vulnerable to residual confounding: the missing indicator method used implies that effects estimated for age or gender are only adjusted for smoking in the 2% individuals that participated in the Dutch Public Health Monitor survey. So, most of these effects will not be adjusted, while the construction method assumes they are adjusted. Using only the Dutch Public Health Monitor sample for fitting a lung cancer model would produce models that are less biased. However, given the low prevalence of lung cancer in this sample, such models would be very imprecise. Accounting for this discrepancy, for use in micro-simulation modelling, we recommend an additional post-calibration step, by increasing the probability of lung cancer during generation of the synthetic population with a factor that makes the prevalence equal to the population marginal.

An important limitation of our modelling approach might be grounded in the fact that we did not include interactions in our models, apart from stratification. As a result, potential interaction become part of the error term, magnifying the unexplained variance. We have observed this in the modelling of household variables, where the relation between personal capital and income was not well described by a simple additive relationship, even when using splines. A more adequate approach could be achieved by means of non-parametric models like random forest, but exporting such models comes with disclosure risk and, therefore, are unfeasible in this context. However, as we experienced manual model selection as a very time consuming task, finding exportable models that automatically include or select important interactions would be a nice avenue to explore in further research.

Due to the large amount of information, challenges were faced in each model as the fit could always be improved. For instance, in the extremes (lowest, highest) of spendable income ranges, the relationship with personal capita is generally curvilinear, while in the middle-income range the relationship is linear. The explanation of this phenomenon at the lower end is partly that in some sectors (like agriculture) high investments (e.g. in land) are needed while income can be low. This relation could be the subject of further methodological and substantive investigations.

We now constructed the synthetic population using the point estimates of the parameters. However, we also exported the covariance matrices of the parameter estimates, so it would be easy to use this to randomly draw parameter sets multiple times and construct sets of synthetic populations, each based on a random draw. These can be used to estimate the influence of the statistical uncertainty in the models on the outcomes of the micro-simulation.

In our approach, all data apart from the seed variables are generated from fitted models. This might be too cautious with regards to disclosure risks. It would be interesting to see whether it is possible to develop a method which mixes real individuals with synthetic individuals and partially synthetic individuals in a manner that has no risk of disclosure.

In this paper, we described the methods we used to construct a synthetic population meant for micro-simulation, based on population wide data liked to data of a large health survey. Although the constructed population does not reflect the original data exactly, we believe the reconstruction is close enough to reality to be suitable for use in micro-simulation. The method can be further improved by using models that include more interactions and transformations of dependent variables with non-normal residuals. It is surely superior to methods constructing initial populations that assume independence between variables. When marginals deviate too much from the available population marginal—mostly for outcomes variables employed later in the construction procedure—calibration can be added to adjust marginals, while maintaining the mutual relations between variables assumed by the models.

## Data Availability

Not applicable.

## Appendix 1: Description of variables

See Table 5.

**Table 5:** The list of variables and their order in the sequential construction of synthetic population

Variable	Definition	Main source	Method of collection	Missing (%)
COROP region	A regional area within the Netherlands	Statistics Netherlands	Definition by SN	0
Level of urbanization	ambient/area address density	Statistics Netherlands	Definition by SN	0
Gender	Indicator	Civic Register	Municipality	0
Age	Age at 31st December 2012	Civic Register	Municipality	0
Source of income	Main source of income in the household	Tax Authorities, DUO	Deducted by SN	0
Spendable income	Percentile of spendable income of the Dutch household	Tax Authorities, DUO	Calculated by SN	0
Personal capital	Percentile of personal capital of the Dutch household	Tax Authorities	Calculated by SN	0
Type of household	Type of household at the beginning of the year	Civic Register	Derived by SN	0
Number of persons in the household	Household size	Civic Register	Derived by SN	0
Ethnic group	Categories based on country of origin	Civic Register	Municipality	0
Education	SOI classification	Statistics Netherlands	Defined by SN	42.3
Smoking	Smoking status	RIVM/statistics Netherlands	Self-assessment	0
BMI	Body mass index	RIVM/statistics Netherlands	Self-assessment	
Physical Activity	Adherence to PA norm	RIVM/Statistics Netherlands	Self-assessment	0
CHD	Probability	Combined health care records*	Calculated by RIVM	0
Stroke	Probability	Combined health care records*	Calculated by RIVM	0
Diabetes	Probability	Combined health care records*	Calculated by RIVM	0
COPD	Probability	Combined health care records*	Calculated by RIVM	0
Pancreatic cancer	Diagnosed	Netherlands Comprehensive Cancer Organization (IKNL)	Reported by doctor	0
Lung cancer	Diagnosed	Netherlands Comprehensive Cancer Organization (IKNL)	Reported by doctor	0

## Appendix 2: Description of models

The starting population to support the models is the complete 2012 Dutch population whose retained characteristics are the seed variables: age (in years), gender and place of residence at the level of COROP code (40-level variable) and level of urbanization. Following exploratory analysis, several observations which characterize all models are notable. All models are stratified by gender. Given that the effects of age on the outcomes vary largely in shape and magnitude, we employ natural cubic splines of age to model these effects with knots at 0, 10, 17, 20, 25, 30, 50, 55, 60, 66, 70, 80 90 and 100 for each stratum in turn. As dependent variables, the percentile of spendable income and the percentile of personal capital are transformed through a linear transformation into *z*-scores. As independent variables, the *z*-scores of the percentile of spendable income and the percentile of personal capital are transformed in natural cubic splines with knots at − 2, − 1, 0, 1 and 2, respectively, for each stratum in turn. The performance of model fit is assessed by tenfold cross-validation.

Model 1 employs main income source of the household (categorical, 14 levels) as dependent variable and the seed variables as independent variables. Stratification is done by gender and COROP region interaction. Eighty multinomial regression models are fitted with main effects of level of urbanization and cubic splines.

Model 2 employs the percentile of spendable income as dependent variable and the seed variables and the main income source of the household as independent variables. A linear regression model is fitted with main effects of COROP region, level of urbanization, cubic splines and main income source of the household.

Model 3 employs the percentile of personal capital as dependent variable and the seed variables, the main income source of the household and the percentile of spendable income as independent variables. A linear regression model is fitted with main effects of the independent variables and natural cubic splines.

Model 4 employs the type of household (categorical, 2 levels) as dependent variable and the seed variables, the main income source of the household, the percentile of spendable income, the percentile of personal capital and the number of persons in the household as independent variables. A multinomial regression model is fitted with main effects of the independent variables and natural cubic splines.

Model 5 employs the number of persons in the household (categorical, 6 levels) as dependent variable and the seed variables, the main income source of the household, the percentile of spendable income and the percentile of personal capital as independent variables. Stratification is done by gender and type of household interaction. A multinomial regression model is fitted with main effects of the independent variables and natural cubic splines.

Model 6 employs the ethnic group as dependent variable (8-level category) and the seed variables, the main income source of the household, the percentile of spendable income, the percentile of personal capital, the number of persons in the household and the type of household as independent variables. Stratification is done by gender and type of household interaction. Eight multinomial regression models are fitted with main effects of the independent variables and natural cubic splines.

Model 7 employs education (categorical, 5 levels) as dependent variable and the seed variables, the main income source of the household, the percentile of spendable income, the percentile of personal capital, the number of persons in the household, the type of household and the ethnic group as independent variables. A multinomial regression model is fitted with main effects of the independent variables and natural cubic splines.

Model 8 employs smoking status (categorical, 4 levels: “never smoker”, “former smoker”, “current smoker” and “heavy smoker”) as dependent variable and the seed variables, the main income source of the household, the percentile of spendable income, the percentile of personal capital, the number of persons in the household, the type of household, the ethnic group and education as independent variables. A multinomial regression model is fitted with main effects of the independent variables and natural cubic splines.

Model 9 employs BMI (continuous) as dependent variable and the seed variables, the main income source of the household, the percentile of spendable income, the percentile of personal capital, the number of persons in the household, the type of household, the ethnic group, education and smoking as independent variables. BMI was measured for individuals older than 18 years. A linear regression model is fitted with main effects of the independent variables and natural cubic splines.

Model 10 employs physical activity (categorical, 2 levels) as dependent variable and the seed variables, the main income source of the household, the percentile of spendable income, the percentile of personal capital, the number of persons in the household, the type of household, the ethnic group, education, smoking and BMI as independent variables. A multinomial regression model is fitted with main effects of the independent variables and natural cubic splines.

Model 11 employs pancreas cancer as dependent variable and the seed variables, the main income source of the household, the percentile of spendable income, the percentile of personal capital, the number of persons in the household, the type of household, the ethnic group, education, smoking, BMI and physical activity as independent variables. A logistic regression model is fitted with main effects of the independent variables and natural cubic splines for the z-scores of percentiles of personal income and personal capital. The missing data indicator approach has been used to deal with the missing values of smoking, BMI and physical activity for those residents not participating to the Health Survey study Missing values of education, smoking, BMI and physical activity within the Health Survey study have a priori been imputed five times; each imputed data sample has been merged with the administrative data of the entire Dutch population.

Model 12 employs lung cancer as dependent variable and the seed variables, the main income source of the household, the percentile of spendable income, the percentile of personal capital, the number of persons in the household, the type of household, the ethnic group, education, smoking, BMI, physical activity and pancreas cancer as independent variables. A logistic regression model is fitted with main effects of the independent variables and natural cubic splines for the z-scores of percentiles of personal income and personal capital. A similar missing data indicator approach has been used to deal with missing values of education, smoking, BMI and physical activity as for the case of pancreas cancer.

Model 13 employs CHD as dependent variable and the seed variables, the main income source of the household, the percentile of spendable income, the percentile of personal capital, the number of persons in the household, the type of household, the ethnic group, education, smoking, BMI, physical activity, lung cancer and pancreas cancer as independent variables. A linear regression model is fitted on the logistic transformation of CHD with main effects of the independent variables and age as factor.

Model 14 employs stroke as dependent variable and the seed variables, the main income source of the household, the percentile of spendable income, the percentile of personal capital, the number of persons in the household, the type of household, the ethnic group, education, smoking, BMI, physical activity, lung cancer, pancreas cancer and CHD as independent variables. A linear regression model is fitted on the logistic transformation of stroke with main effects of the independent variables and age as factor.

Model 15 employs diabetes as dependent variable and the seed variables, the main income source of the household, the percentile of spendable income, the percentile of personal capital, the number of persons in the household, the type of household, the ethnic group, education, smoking, BMI, physical activity, lung cancer, pancreas cancer, CHD and stroke as independent variables. A linear regression model is fitted on the logistic transformation of diabetes with main effects of the independent variables and age as factor.

Model 16 employs COPD as dependent variable and the seed variables, the main income source of the household, the percentile of spendable income, the percentile of personal capital, the number of persons in the household, the type of household, the ethnic group, education, smoking, BMI, physical activity, lung cancer, pancreas cancer, CHD, stroke and diabetes as independent variables. A linear regression model is fitted on the logistic transformation of COPD with main effects of the independent variables and age as factor.
